# Supplementary material for: An exploratory analysis of changes in work values among nurses before and after pregnancy
Source: PLoS One. 2026 Apr 20;21(4):e0347779. doi: 10.1371/journal.pone.0347779 (PMC13094997; doi:10.1371/journal.pone.0347779)
Supplement: S1 Appendix — This is the S1 Appendix: Questionnaire. (DOCX) [file pone.0347779.s001.docx]

**S1 Appendix: Questionnaire**

*(The questionnaire was administered in Japanese and translated into English for this paper.)*

**【Question 1】 About Yourself**

**Age**

Please indicate your age.

____________ years old

**Educational Background**

Please indicate the highest level of professional education you have completed. Check the box(es) that apply. If you are currently enrolled or withdrew mid-course, please choose “Other,” then elaborate on your choice in writing. (Multiple selections allowed)

- University/College (Nursing major)
- Graduate School (Nursing, Master's)
- Graduate School (Nursing, Doctoral)
- Nursing school (2-year program), Nursing school (3-year program), or Integrated 5-year nursing school
- Licensed Practical Nurse (LPN) training school
- Other

**Marital Status**

Please indicate your marital status. Check the box that applies.

- Married
- Unmarried
- Other

**Cohabitants**

Please indicate the family members currently living with you. Check the box(es) that apply. Please answer based on your relationship to them. (Multiple selections allowed)

- No cohabitants
- Spouse/Partner
- Child(ren)
- Father
- Mother
- Spouse's Father
- Spouse's Mother
- Grandparent(s)
- Siblings
- Other

**Number and Age of Children**

How many children do you have?

____________ person(s)

Please indicate the age of your children, starting with the eldest (first child).

- →If 1 was answered:
  - 0 years old / 1 year old / 2 years old / 3 years old / 4 years old / 5 years old / 6 years old
- →If 2 was answered:
  - 0 years old / 1 year old / 2 years old / 3 years old / 4 years old / 5 years old / 6 years old
- →If 3 was answered:
  - 0 years old / 1 year old / 2 years old / 3 years old / 4 years old / 5 years old / 6 years old
- →If 4 was answered:
  - 0 years old / 1 year old / 2 years old / 3 years old / 4 years old / 5 years old / 6 years old
- →If 5 was answered:
  - 0 years old / 1 year old / 2 years old / 3 years old / 4 years old / 5 years old / 6 years old

**Primary Childcare Cooperator**

Is there someone who shares in or cooperates with you in childcare? Check the box that applies.

- Yes
- No

**Childcare Leave**

Please tell us about the maternity/childcare regarding your eldest child (first child). Did you take childcare leave for the birth and care of your eldest child (first child)?

- Took childcare leave
- Did not take childcare leave

**Time Since Return to Work**

How many years and months have passed since you returned to work following maternity/childcare leave for the birth and care of your eldest child (first child)? (Example: 0 years, 3 months)

____________ Year(s) ____________ Month(s)

**Use of Childcare Facilities**

Are you currently using a childcare facility (including daycares, kindergartens, certified *Kodomo-en*, and other community-based childcare services)? Check the box that applies.

- Using an in-hospital nursery
- Using an external childcare facility, not an in-hospital nursery
- Not using one

**Presence of Caregiver**

Is there a family member you primarily provide care for? Check the box that applies.

- Yes
- No

**Your Physical and Mental State**

How is your physical and mental state? Check the box that best applies.

- Both physically and mentally well
- Physically well but mentally unwell
- Mentally well but physically unwell
- Both physically and mentally unwell
- Cannot say (Reason:　　　　　)

**Your Health Status**

How do you feel about your current health status? Check the box that best applies.

- Very good
- Fairly good
- Normal
- Not very good
- Bad

**Concerns about Child's Health/Growth and Development**

Do you have any concerns about your child's development? Check the box that applies.

- Yes
- No

**Optimism/Mental Space**

Do you have time to spend with your child in a relaxed mood? Check the box that applies.

- Yes
- No
- Neutral

**Economic Situation**

We ask about your living situation. Circle the number that applies.

Statement: My economic situation is easy.

Exactly true (5) / True (4) / Neutral (3) / Not true (2) / Not true at all (1)

**Achievement of Ideal Parenting**

Are you currently able to achieve the kind of parenting you consider ideal? Check the box that applies.

- I am achieving it
- I am mostly achieving it
- I am not achieving it much
- I am not achieving it at all

**Social support from family, friends, and significant others**

Thinking about the past week, circle the number that applies.

Very Strongly Agree (7), Strongly Agree (6), Mildly Agree (5), Neutral (4), Mildly Disagree (3), Strongly Disagree (2), Very Strongly Disagree (1)

| **No.** | **Statement** | 7 | 6 | 5 | 4 | 3 | 2 | 1 |
| --- | --- | --- | --- | --- | --- | --- | --- | --- |
| 1 | There is a special person who is around when I  am in need. | 7 | 6 | 5 | 4 | 3 | 2 | 1 |
| 2 | There is a special person with whom I can share  my joys and sorrows. | 7 | 6 | 5 | 4 | 3 | 2 | 1 |
| 3 | My family really tries to help me. | 7 | 6 | 5 | 4 | 3 | 2 | 1 |
| 4 | I get the emotional help and support I need from  my family. | 7 | 6 | 5 | 4 | 3 | 2 | 1 |
| 5 | I have a special person who is a real source of  comfort to me. | 7 | 6 | 5 | 4 | 3 | 2 | 1 |
| 6 | My friends really try to help me. | 7 | 6 | 5 | 4 | 3 | 2 | 1 |
| 7 | I can count on my friends when things go wrong. | 7 | 6 | 5 | 4 | 3 | 2 | 1 |
| 8 | I can talk about my problems with my family. | 7 | 6 | 5 | 4 | 3 | 2 | 1 |
| 9 | I have friends with whom I can share my joys  and sorrows. | 7 | 6 | 5 | 4 | 3 | 2 | 1 |
| 10 | There is a special person in my life who cares  about my feelings. | 7 | 6 | 5 | 4 | 3 | 2 | 1 |
| 11 | My family is willing to help me make decisions. | 7 | 6 | 5 | 4 | 3 | 2 | 1 |
| 12 | I can talk about my problems with my friends. | 7 | 6 | 5 | 4 | 3 | 2 | 1 |

**【Question 2】 About Work Experience**

**Occupation**

Please indicate your current occupation. Check the box that applies.

- Registered Nurse
- Midwife
- Public Health Nurse
- Licensed Practical Nurse
- Other

**Years of Experience**

How many years of nursing experience do you have? Please answer with the year you first started working as a nursing professional as "1st year".

____________ year(s)

**Current Workplace**

Please indicate your current workplace. Check the box that applies.

- Hospital
- Clinic with beds
- Clinic without beds
- Elderly Care/Welfare Facility
- Visiting Nurse Service
- Daycare/Kindergarten
- General Company
- Nursing Training Institution
- Other

**Years of Service at Current Workplace**

How many years have you been with your current employer? Please answer with the year you started working there as "1st year".

____________ year(s)

**Employment Status**

Please indicate your current employment status. Check the box that applies.

- Regular employee (Full-time)
- Regular employee (Short-time work)
- Non-regular employee (Contract, temporary, part-time, etc.)
- Other

**Work Schedule**

Please indicate your current work schedule. Check the box that applies.

- Day shift only
- Night shift only
- Shift work (Rotation)
- Other

**Average Actual Working Hours Per Day (Including Overtime)**

Please indicate your average weekly working hours, including overtime, that you have actually worked recently. Check the box that applies.

- 1–25 hours
- 26–35 hours
- 36–45 hours
- 46–55 hours
- 56 hours or more

**Workplace Social Support**

We ask about the people around you. Circle the one that best applies.

**How easily can you talk to the following people?**

| **Person** | **Very much (4)** | **Quite a bit (3)** | **Somewhat (2)** | **Not at all (1)** |
| --- | --- | --- | --- | --- |
| Supervisor | 4 | 3 | 2 | 1 |
| Colleagues | 4 | 3 | 2 | 1 |

**When you are in trouble, to what extent can you rely on the following people?**

| **Person** | **Very much (4)** | **Quite a bit (3)** | **Somewhat (2)** | **Not at all (1)** |
| --- | --- | --- | --- | --- |
| Supervisor | 4 | 3 | 2 | 1 |
| Colleagues | 4 | 3 | 2 | 1 |

**To what extent will the following people listen if you consult with them about your personal problems?**

| **Person** | **Very much (4)** | **Quite a bit (3)** | **Somewhat (2)** | **Not at all (1)** |
| --- | --- | --- | --- | --- |
| Supervisor | 4 | 3 | 2 | 1 |
| Colleagues | 4 | 3 | 2 | 1 |

**Work Values**

How important are the following items to you in working as a nursing professional? For each question, choose and circle one number from 1 to 5.

| **No.** | **Item** | very important | quite important | somewhat important | not very important | not important at all |
| --- | --- | --- | --- | --- | --- | --- |
| 1 | To grow as licensed practical nurses, registered nurses, midwives, and public health nurses | 5 | 4 | 3 | 2 | 1 |
| 2 | To enhance practical skills as licensed practical nurses, registered nurses, midwives, and public health nurses | 5 | 4 | 3 | 2 | 1 |
| 3 | Learning new knowledge and skills | 5 | 4 | 3 | 2 | 1 |
| 4 | Earn an above-average salary | 5 | 4 | 3 | 2 | 1 |
| 5 | Working as a full-time employee rather than a part-time employee | 5 | 4 | 3 | 2 | 1 |
| 6 | Guaranteed long-term employment | 5 | 4 | 3 | 2 | 1 |
| 7 | Helping as many people as possible by working as licensed practical nurses, registered nurses, midwives, and public health nurses | 5 | 4 | 3 | 2 | 1 |
| 8 | Helping patients and clients by working as licensed practical nurses, registered nurses, midwives, and public health nurses | 5 | 4 | 3 | 2 | 1 |
| 9 | Contributing to the team and organization as licensed practical nurses, registered nurses, midwives, and public health nurses | 5 | 4 | 3 | 2 | 1 |
| 10 | Being respected as licensed practical nurses, registered nurses, midwives, and public health nurses | 5 | 4 | 3 | 2 | 1 |
| 11 | Receive high marks from patients and clients | 5 | 4 | 3 | 2 | 1 |
| 12 | To be respected by junior stuff | 5 | 4 | 3 | 2 | 1 |

**Work Values Before Pregnancy**

Please recall the time before the pregnancy of your eldest child (first child). How important were the following items to you at that time? For each question, choose and circle one number from 1 to 5.

| **No.** | **Item** | very important | quite important | somewhat important | not very important | not important at all |
| --- | --- | --- | --- | --- | --- | --- |
| 1 | To grow as licensed practical nurses, registered nurses, midwives, and public health nurses | 5 | 4 | 3 | 2 | 1 |
| 2 | To enhance practical skills as licensed practical nurses, registered nurses, midwives, and public health nurses | 5 | 4 | 3 | 2 | 1 |
| 3 | Learning new knowledge and skills | 5 | 4 | 3 | 2 | 1 |
| 4 | Earn an above-average salary | 5 | 4 | 3 | 2 | 1 |
| 5 | Working as a full-time employee rather than a part-time employee | 5 | 4 | 3 | 2 | 1 |
| 6 | Guaranteed long-term employment | 5 | 4 | 3 | 2 | 1 |
| 7 | Helping as many people as possible by working as licensed practical nurses, registered nurses, midwives, and public health nurses | 5 | 4 | 3 | 2 | 1 |
| 8 | Helping patients and clients by working as licensed practical nurses, registered nurses, midwives, and public health nurses | 5 | 4 | 3 | 2 | 1 |
| 9 | Contributing to the team and organization as licensed practical nurses, registered nurses, midwives, and public health nurses | 5 | 4 | 3 | 2 | 1 |
| 10 | Being respected as licensed practical nurses, registered nurses, midwives, and public health nurses | 5 | 4 | 3 | 2 | 1 |
| 11 | Receive high marks from patients and clients | 5 | 4 | 3 | 2 | 1 |
| 12 | To be respected by junior stuff | 5 | 4 | 3 | 2 | 1 |

**Open-Ended Question**

Have you ever felt that your values have changed since you became pregnant with your first child? If so, what values do you think have changed and how? Please freely write about these points.

Thank you for your cooperation.
