# Supplementary material for: An exploratory analysis of changes in work values among nurses before and after pregnancy
Source: PLoS One. 2026 Apr 20;21(4):e0347779. doi: 10.1371/journal.pone.0347779 (PMC13094997; doi:10.1371/journal.pone.0347779)
Supplement: Attachment — Submitted filename: review comments new.pdf [file pone.0347779.s004.pdf]

REVIEW COMMENTS

The authors have addressed almost all the comments raised previously. however, the authors need to address a few minor issues in relation to their ethical consideration and methodology.

ETHICAL CONSIDERATION

1. LINE 147; The last sentence states that the study does not include minors. That was very clear; however, the authors could briefly state why minors are not included to avoid queries (e.g., "As all participants were required to be nurses and parents, no minors were eligible for inclusion.").

ANALYSIS METHOD

1. I suggest the authors consider revising the heading "Analysis method" to "Data Analysis" as a top-level subsection. This sounds more standard.

2. In the qualitative part, the authors mention that "validity was assessed by two other researchers." That was perfect; however, I suggest they use the standard qualitative term "trustworthiness" or "rigor" in their description and specify the process. This strengthens the claim.

3. The author's description ("inductively grouped... abstract subcategories and categories…") is essentially ‘inductive thematic analysis.’ I therefore suggest that the authors be clear and use the term inductive thematic analysis. This term adds methodological recognition to their work.
