# Supplementary material for: An exploratory analysis of changes in work values among nurses before and after pregnancy
Source: PLoS One. 2026 Apr 20;21(4):e0347779. doi: 10.1371/journal.pone.0347779 (PMC13094997; doi:10.1371/journal.pone.0347779)
Supplement: Attachment — Submitted filename: REVIEWERS COMMENTS.pdf [file pone.0347779.s006.pdf]

The authors satisfactorily responded to all comments. I recommend that the manuscript be considered for publication.
