# Supplementary material for: An exploratory analysis of changes in work values among nurses before and after pregnancy
Source: PLoS One. 2026 Apr 20;21(4):e0347779. doi: 10.1371/journal.pone.0347779 (PMC13094997; doi:10.1371/journal.pone.0347779)
Supplement: Attachment — Submitted filename: Response_to_reviewer_20251223.docx [file pone.0347779.s005.docx]

Responses to Reviewers’ Comments

Paper Title: An exploratory analysis of changes in work values among nurses before and after pregnancy

Journal Name: *PLOS ONE*

We would like to express our sincere gratitude for your review of our paper and your valuable and constructive feedback. All comments have been extremely helpful in improving the quality and transparency of this study, and we have thoroughly revised the manuscript based on your suggestions. Our responses to the comments are provided below. The revised sections are highlighted in red within the manuscript.

**Editor Comments**

**Comment 1:** Clarify and strengthen the theoretical framework surrounding work values and their transformation, ensuring that the conceptual basis is well-defined and supported by relevant literature.

Response 1:

Thank you for your valuable comment. We have significantly revised the Introduction and Discussion sections to strengthen the theoretical background regarding work values and their transformation. Based on previous research, we have enriched the discussion of the impact of life events (particularly pregnancy and childcare) on career values, clarifying the conceptual foundation of this study.

Revisions: Introduction and Discussion sections

**Comment 2:** Revise the methodology section to enhance transparency and rigor, particularly in the qualitative component. The procedures for data collection, coding, and analysis need to be described in greater detail.

Response 2:

Thank you for your advice. We have revised the entire Methods section and added more details, especially about the procedures for collecting and analyzing qualitative data. This includes the method for collecting qualitative data (open-ended questions), the number of analysts, the process for deriving categories and subcategories, and the fact that no software was used for analysis.

Revision: Methods

**Comment 3:** Improve statistical reporting by including effect sizes, confidence intervals, and model diagnostics. This will allow for a more robust interpretation of the findings.

Response 3:

Based on your comment, we have added effect sizes (Cohen's d) and 95% confidence intervals to the statistical analysis results. We have also added information about GEE model diagnostics to the methodology to strengthen the robustness of the interpretation of the results.

Revision: Methods/Results sections

**Comment 4:** Enhance the integration of qualitative and quantitative findings to justify the use of a mixed-methods approach. Currently, the connection between both strands of data is weak and underdeveloped.

Response 4:

Reviewers #1 and #4 made similar comments, so we reframed this study as "primarily a quantitative design with supplemental qualitative insights" and significantly strengthened the integration of qualitative and quantitative findings in the discussion.

Revision: Discussion section

**Comment 5:** Provide access to anonymized data or offer a more detailed justification for the restrictions imposed. The current data availability statement does not meet PLOS ONE’s transparency standards.

Response 5:

We have revised our data availability statement to comply with *PLOS ONE* policy. Specifically, we now state, "All relevant data are within the manuscript and its Supporting Information files."

**Comment 6:** Conduct a thorough language revision to improve clarity, grammar, and readability. While the manuscript is generally intelligible, several sections contain awkward phrasing and typographical errors.

Response 6:

Following your suggestion, the language of the manuscript was reviewed and enhanced by a professional English editing service. Further, we focused on correcting grammatical errors, ensuring clarity of expression, refining the academic tone, and improving the overall fluency and readability of the text.

**Reviewer #1 Comments**

**Comment 1:** The use of a retrospective cross-sectional survey is very practical given the difficulty of longitudinal tracking in this population. The paired t-tests and Generalized Estimating Equations (GEE) are suitable for analyzing within-subject changes and identifying associated factors. Your study also employed the use of established scales for work values (Shortened Nurses' Work Values Scale), social support (MSPSS), and workplace support, which enhanced measurement reliability. Again, power analysis using GPower was clearly reported, with a sample size of 199 meeting the requirements for regression analysis with adequate power.

Response 1:

Thank you very much for your understanding of the strengths and characteristics of this study. Your insightful suggestions have significantly improved this manuscript.

**Comment 2:** The title of the manuscript makes it look like it is a mixed-methods paper; however, the execution of the study relies heavily on quantitative methods/analysis. There is very limited or no integration of qualitative methods. Can you explain why this is the case? There are no clear qualitative research questions or a rationale for a mixed methods approach. Again, the qualitative analysis is described with no mention of coding procedures and analysis. I recommend that you substantiate the mixed-methods claim by elaborating the qualitative design (sampling, coding, integration strategy) or reframing the study as a primarily quantitative design with supplemental qualitative insights. You would have to justify the use of the mixed methods approach.

Response 2:

Thank you for your valuable comments. We deeply regret that the mixed methods approach of this study was unclear.

1. Changes to the research design: Based on your comments, we restructured this study as a "primarily quantitative design with supplemental qualitative insights" and clarified the reasons for its adoption in the Introduction and Methods sections.

2. Clarification of the qualitative research objectives: We clearly stated that the qualitative questions (open-ended text) intended to delve deeper and contextualize the quantitative results.

3. Strengthening the methodology: We have enhanced transparency by providing detailed descriptions of the qualitative data sampling, analysis procedures, and integration strategy with the quantitative results.

Revisions: Introduction, lines 81-84 / Methods, lines 88-90, 273-289

**Comment 3:** The quantitative findings of the study are significant; however, there is no clear demonstration as to how the qualitative findings reinforce or contrast with the quantitative results. The research topic indicates that the paper is a mixed-method, but there is insufficient qualitative information to back or justify this method.

Response 3:

Thank you for your comment. In the discussion section, we specifically integrated how the qualitative findings supported the quantitative results (e.g., a decline in prestige work values) or contextualized the underlying personal and professional conflicts. This clearly demonstrated the value of using qualitative findings.

Revisions: The entire Discussion section

**Comment 4:** On statistical analysis, the manuscript lacks detail on the correlation structure used in GEE and whether model diagnostics were performed. You would also need to justify the rationale for the effect size selected for the power analysis.

Response 4:

Based on your comments, we have revised the Methods section.

1. GEE details: We have added information about the exchangeable structure used in the GEE analysis and the model diagnostics (QIC comparison) that supported the adoption of that structure.

2. Rationale for power analysis: We have clarified that the effect size (0.15) used in the power analysis was selected based on previous research, and have justified this rationale.

Revisions: Methods, lines 263-269 and 119-120

**Comment 5:** Please review the abstract for coherence and consistency with the findings of the paper. You may consider being brief with respect to the methodology and reducing the word count.

Response 5:

Based on your comments, we have revised the abstract. We have removed redundant technical terms and simplified the methodology description. Additionally, we have ensured consistency with the findings of the paper by briefly including key quantitative findings and supporting important qualitative insights.

Revisions: Throughout the Abstract

**Comment 6:** The discussion could be expanded to consider implications for nursing policy and workforce retention strategies in Japan, especially in light of the aging population and gender equity challenges.

Response 6:

Thank you for your suggestion. We have added a new subsection, "Policy and Practice Implications," to the Discussion section to delve deeper into the policy and practice implications that changes in work values have on nurse retention and career support in the context of Japan's aging population and the promotion of gender equality.

Revision: Discussion, lines 511-535

**Comment 7:** Consider including a limitations subsection that explicitly addresses recall bias, generalizability, and the cross-sectional design.

Response 7:

Based on your comments, we have significantly revised the "Study limitations" subsection at the end of the discussion. We now clearly discuss the recall bias resulting from the retrospective cross-sectional design, the limitations on generalizability resulting from web-based sampling, and how to address these issues.

Revision: Study limitations, lines 537-561

**Reviewer #2 Comments**

Dear Authors,

Thank you for your submission. Your study addresses an important and underexplored topic: the transformation of work values among Japanese nurses before and after pregnancy. However, several critical issues must be addressed to improve the manuscript’s scientific rigor, clarity, and overall contribution. Below are detailed comments and suggestions organized by section:

Response:

Thank you very much for understanding the strengths of this study. Your advice has helped us further improve this manuscript.

**Abstract**

**Comment:** The abstract is overly dense and includes methodological jargon that may not be accessible to all readers. Consider simplifying and clarifying the structure. The phrase “pioneering mixed-methods study” is overstated given the limitations of the design and analysis. The conclusion should better reflect the limitations and avoid generalizations not supported by the data.

Response:

Based on your comments, we have made significant revisions to the abstract. Specifically, we have avoided excessive methodological jargon and written it succinctly, focusing on the core of the study and its main results. Furthermore, we have removed the overly explicit phrase "pioneering mixed-methods study." We have also revised the conclusion to be more cautious and evidence-based, presenting the main findings while keeping in mind the limitations of the design.

**Introduction**

**Comment:** The literature review is superficial. Key concepts such as “work values” and “value transformation” are not sufficiently defined or contextualized. The rationale for focusing on Japanese nurses is clear, but the global relevance of the findings is overstated. The introduction would benefit from a clearer articulation of the research questions and hypotheses.

Response:

Based on your comments, we have revised the Introduction to improve its logical flow and eliminate redundancies.

1. Conceptual clarification: We conducted a thorough literature review of "work values" and "changes in values," establishing clear definitions and a solid theoretical foundation of these key concepts.

2. Global relevance: While focusing the discussion on the context of Japanese nurses, we adjusted the format to discuss universal implications of the results (e.g., issues related to balancing work and family life) and avoided overgeneralization.

3. Research questions and hypotheses: We clearly stated the quantitative research questions. However, as you acknowledge, this research area—exploring changes in work values among mothers from pre-pregnancy to post-natal care after childbirth and childcare—including the factors influencing these changes, is still unexplored. As this study was positioned as an exploratory analysis, it was difficult to formulate clear hypotheses.

Revisions: The entire Introduction section

**Methods**

**Comment:** The retrospective design is problematic. The authors acknowledge this but do not provide sufficient mitigation strategies. The use of G*Power to justify sample size is appropriate, but the choice of effect size (0.15) and power (0.95) should be justified in the context of prior literature. The description of the survey instrument lacks detail. How were the questions validated? Were they piloted? The qualitative component is inadequately described. What was the coding process? How were themes derived? Was intercoder reliability assessed?

Response:

The methods section was thoroughly revised.

1. Recall bias: In addition to the study limitations section, measures to enhance the reliability of retrospective data (e.g., clarity of questions, facilitation of recall based on specific life events) were added to the Methods section. (Lines 100-109)

2. Power rationale: The selection of effect size (0.15) and power (0.95) were justified based on previous research. (Lines 119-120)

3. Questionnaire description: For scales (e.g., Shortened Nurses' Work Values Scale, MSPSS), the original development papers were cited and descriptions of the scale structure, subscales, and reliability (Cronbach's alpha) used in this study were added. Since these scales have been validated in previous studies, no pilot studies or revalidation was conducted in this study. Furthermore, the variables described in the Methods section were added to the supplementary material (S1 Appendix) to improve readability and clarify how they were used in the questionnaire.

4. Qualitative data analysis: We have strengthened the transparency of our qualitative data analysis by providing detailed descriptions of the analytical procedures and methods, the number of analysts, and the procedures for verifying the validity of the analysis among researchers. (Lines 273-289)

**Analysis of quantitative variables**

**Comment:** The use of paired t-tests is simplistic and does not account for potential confounders. The application of Generalized Estimating Equations (GEE) is appropriate but poorly explained. What correlation structure was used? Were model diagnostics performed? The results section lacks effect sizes and confidence intervals, which are essential for interpreting the magnitude and precision of findings. There is no discussion of missing data or how it was handled.

The presentation of results is fragmented. Tables are referenced but not included in the review document. The finding that only prestige work values decreased significantly is interesting but not explored in depth. The GEE results are presented without sufficient interpretation. What do these associations mean in practical terms?

Response:

The statistical analysis, results, and discussion sections have been significantly revised.

1. Role of t-tests and GEE: We clarified that t-tests were used to demonstrate simple descriptive changes, and that GEE was the primary analysis, including potential confounders. (Lines 227-232, 244-246)

2. Details and reporting of t-test and GEE results: We added details on correlation structures and model diagnostics. Additionally, we added effect sizes and 95% confidence intervals to all statistical results. Furthermore, we provided additional textual explanations for significant results presented in tables. (Results section)

3. Handling of missing values: There were no missing values for any variables, and this was clearly stated. (Lines 242-243)

4. Interpretation of results: We integrated the "decrease in prestige work values" and other results with qualitative findings (e.g., work-life balance, time constraints) and added an interpretation in the discussion section that delves deeper into their practical implications (e.g., decreased interest in promotion). (Results and Discussion sections)

**Qualitative descriptive analysis of descriptive statistics**

**Comment:** The qualitative findings are superficial. There is no evidence of methodological rigor (e.g., thematic saturation, triangulation). Quotes from participants are not included, which limits the reader’s ability to assess the validity of the interpretations. The integration of qualitative and quantitative findings is weak. Consider using a joint display or narrative synthesis.

Response:

The Methods, Results, and Discussion sections have been revised.

1. Regarding the Study Design: As noted in the limitations, qualitative analysis is limited to open-ended responses and does not provide the deep insights of in-depth interviews. Therefore, this study adopted a primarily quantitative design with supplemental qualitative findings integrated.

2. Regarding Participant Quotations and Strengthened Integration: Examples of data from representative participants supporting each theme are provided in the "Examples of the Included Data" column of Results Table 5 (pp. 29-31). In the discussion, we have also strengthened the integration of qualitative findings by adding additional information on how participants actually expressed themselves, rather than just categories and subcategories.

**Discussion**

**Comment:** The discussion overstates the implications of the findings. The limitations of the design and analysis should be more prominently acknowledged. The authors should avoid normative statements such as “Understanding these shifts is crucial…” unless supported by evidence. The discussion lacks engagement with broader literature on gender, work-life balance, and nursing retention.

Response:

The entire Discussion section has been revised.

1. Clarification of Limitations: The limitations of the design and analysis have been discussed in more detail in the “Study Limitations” subsection.

2. Modification of Prescriptive Statements: Prescriptive statements such as "Understanding these shifts is crucial..." have been revised to be more conservative.

3. Addition of Subsections: A "Policy and Practice Implications" subsection has been added to strengthen the discussion of international literature on gender, work-life balance, and nurse retention, extending the relevance of the study beyond the Japanese context.

**Ethics and Data Availability**

**Comment:** The ethics statement is thorough and appropriate. However, the data availability is insufficient. PLOS ONE requires full data transparency. The authors must provide anonymized datasets or justify restrictions more clearly.

Response:

We have revised our data availability statement to comply with *PLOS ONE* policy. Specifically, we now state, "All relevant data are within the manuscript and its Supporting Information files."

**Language and Style**

**Comment:** The manuscript is generally intelligible but contains awkward phrasing and grammatical inconsistencies. A professional language edit is recommended. Examples: “not very import” (likely a typo), “prestige work values showed a significant decrease in the mean” (awkward phrasing).

Response:

The language throughout the manuscript was thoroughly revised through a professional English editing service.

**Reviewer #3 Comments**

**Comment 1:** In the abstract, the results section speaks only to the quantitative findings. Briefly mention at least one key qualitative insight from the results.

Response 1:

Thank you for your comment. we have briefly added key qualitative insights to the results section of the abstract that contextualize the quantitative changes (e.g., the decrease in prestige work value).

**Comment 2:** Line 64- The sentence starting at line 64 is virtually a repetition of the sentence starting at line 49. Consider combining the two paragraphs or improving their flow.

Response 2:

As you pointed out, there was some overlap. Therefore, we reviewed the relevant paragraphs and consolidated or reworded them to improve the flow.

**Comment 3:** Line 180- keep the same referencing style

Response 3:

Based on your suggestions, we have made the citation style of the references consistent throughout the manuscript.

**Comment 4:** Lines 195 to 197- How qualitative data was collected is not clear. Authors should indicate the data collection method used, whether by face-to-face interview or focus group discussion. Which tool was used to collect the data? Was it by audio recording or text? The qualitative aspect of data collection lacks rigor. It does not address questions such as “what type of data analysis was used? Was it content or thematic analysis?”, “Who did the coding?”, “How was the coding done?” and “Whether any software was used in managing the data?” among others. Authors should rectify this.

Response 4:

We strengthened the transparency of our qualitative data collection and analysis processes.

1. Collection Method: We clearly stated that qualitative data were collected in text format through open-ended questions within an online survey. (Lines 219-220)

2. Analytical Rigor: We significantly strengthened the transparency of our qualitative data analysis. We provided detailed descriptions of the analytical method used (qualitative descriptive analysis), the fact that no software was used, the analytical procedures, the number of analysts, and the procedures for inter-researcher validation of the analysis. (Lines 273-289)

**Comment 5:** Line 90- The Authors mentioned Retrospective recall bias as part of the limitations. Authors should go beyond citing Thigpen and Hipp to indicate in the methods section, steps taken to mitigate this, to improve the validity of the study approach.

Response 5:

Thank you for your comment. We have added details about specific steps to mitigate recall bias (e.g., establishing a clear baseline of pre-pregnancy) in the Methods section as well as the Study Limitations subsection. (Lines 100-109)

**Comment 6:** Line 98- In Japan, preschool covers a wide age range; therefore, specifying the age range of their first child will be more precise.

Response 6:

Based on your feedback, we have revised the statement regarding participants' first children in the Methods section to specify a more accurate age range. (Lines 114-115)

**Comment 7:** Variables were well listed; however, to improve readability, I suggest the authors use a table either in the manuscript or on a different sheet as a supplementary document to capture the variables listed.

Response 7:

Thank you for your suggestion. We have added Supporting Information (S1 Appendix) to the variables described in the Methods to improve readability and clarify how they were used in the questionnaire. (Lines 155-156)

**Comment 8:** Line 275- insert the necessary figures or remove the phrase.

Response 8:

Thank you for pointing this out. In accordance with the submission guidelines, the figures were submitted as separate files. We have now removed the phrase as suggested to avoid any confusion and to ensure the manuscript's consistency.

**Comment 9:** Either by Omission or misunderstanding on my part, I did not find an explanation from the qualitative data suggesting why there is a drop in prestige. Authors should indicate if this was just a feeling of the participants or otherwise.

Response 9:

Thank you for your comment. In the discussion section, We clarified that the decline in prestige work values (quantitative result) is supported by specific themes extracted from the qualitative analysis, such as the category "My life became centered on my children and family," and particularly its subcategories, "Children and family come first," and "My thoughts and actions at work have changed (negative)." Furthermore, to further strengthen the integration of qualitative findings, we explained that this is supported by data that directly express constraints on status and career progression, such as "It takes time to build a career with reduced hours." We clarified that this is not simply a sentiment, but a structural shift in priorities that accompanies a change in role. (Lines 433-443)

**Reviewer #4 Comments**

This manuscript addresses an important and underexplored topic. However, some revisions are needed to improve clarity, structure, and analytical depth.

Response:

Thank you very much for understanding the strengths of this study. Your advice has further helped us improve this manuscript.

**Comment 1:** Study Design and Recall Bias: The use of a retrospective cross-sectional design is acknowledged, but here recall bias remains a concern. Authors should further justify the reliability of recalling pre-pregnancy work values and discuss how this may have influenced results.

Response 1:

We justified the use of a retrospective design and added measures to mitigate recall bias to the Methods (lines 100-109). We also provided a more in-depth discussion of the impact this bias may have had on the results in the Study Limitations subsection in the Discussion (lines 538-545).

**Comment 2:** Methodological Detail: Please clarify the correlation structure assumed in the GEE model

Response 2:

We added the exchangeable structure used in the GEE analysis and the model diagnostics (QIC comparison) that supported the adoption of that exchangeable structure. (Lines 263-269)

**Comment 3:** Sample Representation: The sampling process is not fully explained. How samples were selected and how was the web survey done?

Response 3:

We added details to the Methods regarding the sampling method, participant recruitment method, and web survey implementation procedures (use of a research company, screening process) (lines 121-130).

**Comment 4:** Ethics Section: Clarify whether consent was implied or explicitly obtained through digital checkboxes.

Response 4:

We clarified in the ethics section that consent from participants was explicitly obtained through a digital checkbox (lines 141-143).

**Comment 5:** Qualitative Analysis: The qualitative analysis section needs clearer explanation of the coding process (e.g., number of coders, inter-rater agreement, any use of software).

Response 5:

The qualitative analysis section was enhanced by including details about the number of analysts, the procedures for cross-researcher validation of the analysis, and the fact that no software was used for data analysis (lines 273-289).

**Comment 6:** The inclusion of participant IDs in Table 5 is appreciated. If feasible, consider adding a brief note in the methods to explain that these IDs

Response 6:

As suggested, we have added more detail to the Methods section. We have clarified that participant IDs in Table 5 were assigned to facilitate the integration of qualitative and quantitative data and do not contain personally identifiable information. (Lines 287-289)

**Comment 7:** Integration of Mixed Methods: The mixed-methods design would be stronger with explicit integration—how do the qualitative findings confirm, complement, or contradict quantitative results?

Response 7:

We reframed this study as a "primarily quantitative design with supplemental qualitative insights" and strengthened the integration of qualitative and quantitative findings in the Discussion section.

**Comment 8:** Data Availability: PLOS ONE requires publicly available data. Data availability statement is incomplete and non-compliant with PLOS ONE policy.

Response 8:

We have revised our data availability statement to conform with *PLOS ONE* policy. Specifically, we now state, "All relevant data are within the manuscript and its Supporting Information files."

**Comment 9:** Discussion: Expand on implications for hospital policy and management beyond Japan.

Response 9:

A new subsection, "Policy and Practice Implications," was added to the Discussion section to explore the implications of the study's findings for international hospital policy and management, not just for Japanese nurses. (lines 511-535)

**Comment 10:** References: DOI links should be active and complete.

Response 10:

We have reviewed the entire reference list and confirmed that all DOI links are active and in their complete form.

Once again, we would like to thank you for your valuable comments and guidance. We believe that these revisions have made the manuscript stronger and clearer, meeting *PLOS ONE*'s publication standards.
