# Supplementary material for: An exploratory analysis of changes in work values among nurses before and after pregnancy
Source: PLoS One. 2026 Apr 20;21(4):e0347779. doi: 10.1371/journal.pone.0347779 (PMC13094997; doi:10.1371/journal.pone.0347779)
Supplement: Attachment — Submitted filename: Response_to_reviewer_20260209.docx [file pone.0347779.s007.docx]

Responses to Reviewers’ Comments

Paper Title: An exploratory analysis of changes in work values among nurses before and after pregnancy

Journal Name: *PLOS ONE*

We would like to express our sincere gratitude for your further review of our paper and for your valuable and constructive feedback. We are encouraged by the positive assessment of our revisions. We have addressed the remaining points as suggested, which we believe have further polished the manuscript. Our responses to the comments are provided below. The revised sections are highlighted in red in the manuscript.

**Reviewer #1 Comments**

**Comment:** Congratulations on making significant improvements to the manuscript and responding to all reviewer and editor comments. This has made the manuscript very strong, and it now has a clearer theoretical grounding of work values and value transformation. There is equally a much improved qualitative analysis section. the results section and the discussion section are well written and coherent.

**Response:** Thank you very much for your kind words and for recognizing the improvements made to our manuscript. We truly appreciate the time and effort you have dedicated to providing such constructive feedback throughout the review process.

**Comment:** However, here are a few suggestions

1. in your abstract, as your keyword, you use the word/phrase "nursing stuff." It is not clear if that is what you really want to write. The actual word should be "nursing staff." What do you think? Please clarify and correct.

**Response 1:** Thank you very much for pointing out this typographical error. We have corrected "nursing stuff" to "nursing staff" in the keywords section of the abstract. We apologize for the oversight.

Revisions: Abstract (Keywords)

**Comment:** 2. Your transitions from paragraph to paragraph also appear to be abrupt. Please double-check and ensure that there is a flow between the paragraphs and that they transition smoothly.

**Response 2:** We appreciate this suggestion. We have reviewed the entire manuscript and added transitional phrases or adjusted the opening sentences of the paragraphs to ensure a smoother logical flow and better connectivity between sections, particularly in the Introduction and Discussion.

Revisions: Throughout the manuscript.

**Comment:** 3. In the qualitative analysis, it will be appropriate to put the supporting statements in quotation marks (i.e., "...") and italicize them, alongside the participant code or pseudonym.

**Response 3:** Thank you for this specific guidance on formatting. We have revised the qualitative findings section so that all supporting qualitative statements (quotes) are now enclosed in quotation marks and italicized. We have also ensured that participant codes are consistently placed alongside each quote.

Revisions: Results (Qualitative findings), Discussion sections.

**Comment:** 4. some small language copyediting of the entire manuscript will do.

**Response 4:** We appreciate the suggestion. The entire manuscript has undergone further language copyediting to improve clarity, grammar, and overall readability.

Revisions: Throughout the manuscript.

**Reviewer #3:**

**Comment:** [REVIEW COMMENTS] The authors have addressed almost all the comments raised previously. however, the authors need to address a few minor issues in relation to their ethical consideration and methodology.

**Response:** Thank you for your valuable feedback. In response to your suggestion, we have further refined the Ethical Considerations and Methodology sections to ensure greater clarity and transparency.

**Comment:** [ETHICAL CONSIDERATION] 1. LINE 147; The last sentence states that the study does not include minors. That was very clear; however, the authors could briefly state why minors are not included to avoid queries (e.g., "As all participants were required to be nurses and parents, no minors were eligible for inclusion.").

**Response:** Thank you for this helpful suggestion. To clarify, we have added the following sentence to the "Ethical Considerations" section: "As all participants were required to be licensed nurses and parents, no minors were eligible for inclusion."

Revisions: Ethical considerations subsection in the Methods section.

**Comment:** [ANALYSIS METHOD] 1. I suggest the authors consider revising the heading "Analysis method" to "Data Analysis" as a top-level subsection. This sounds more standard.

**Response:** We agree that "Data Analysis" is more standard. We have updated the heading accordingly.

Revisions: Methods section (subheading).

**Comment:** [ANALYSIS METHOD] 2. In the qualitative part, the authors mention that "validity was assessed by two other researchers." That was perfect; however, I suggest they use the standard qualitative term "trustworthiness" or "rigor" in their description and specify the process. This strengthens the claim.

**Response:** Thank you for this suggestion. We used the terms "trustworthiness" and "rigor" instead of "validity" and provided a detailed explanation of the peer debriefing process to demonstrate the rigor of the qualitative analysis.

Revisions: Data Analysis subsection in the Methods section

**Comment:** [ANALYSIS METHOD] 3. The author's description ("inductively grouped... abstract subcategories and categories…") is essentially ‘inductive thematic analysis.’ I therefore suggest that the authors be clear and use the term inductive thematic analysis. This term adds methodological recognition to their work.

**Response:** We have revised the text to explicitly state that we employed "inductive thematic analysis." We agree that using this established term provides better methodological recognition for our qualitative approach.

Revisions: Throughout the manuscript.

**Reviewer #4:**

**Comment:** The manuscript now largely meets the required standards. Only minor wording refinements are needed to avoid unintended longitudinal implications, along with a brief clarification on the direction of recall bias. No major methodological issues remain.

**Response:** We are grateful for your positive assessment. We have addressed your remaining points as follows:

1. **Wording Refinements:** To avoid any implication of a prospective longitudinal design, we have consistently used terms such as "retrospective assessment" or "perceived changes" throughout the manuscript to clarify the cross-sectional nature of our data collection.
2. **Recall Bias:** We have added a discussion in the "Limitations" section regarding recall bias. We noted that while retrospective reports can be influenced by current circumstances, major life events like pregnancy often serve as "temporal anchors" that may improve recall accuracy, while acknowledging that social desirability bias could still play a role.

Revisions: Methods, Discussion (Limitations) sections.

Once again, we would like to thank you for your valuable comments and guidance. We believe that these revisions have made the manuscript stronger and clearer, meeting *PLOS ONE*'s publication standards.
